# Ventilation

**Published:** 1881-02

**Authors:** 


HALL'S

### JOURNAL OF HEALTH

AND

MISCELLANY.

E. H. GIBBS, A. M., M. D. - - EDITOR.

Founded in 1854, and published Monthly without Interruption since that Time.

### VENTILATION.

WE BREATHE in air, and we breathe out carbonic acid gas. This process goes on ceaselessly, from birth to death. Air is an invisible substance in which we live, as fish live in water. Both air and water are subject to contaminations that render them dangerous to health. Air consists of about one part of oxygen and four parts of nitrogen, with traces of other elements. As air is drawn to the lungs, its oxygen combines with the carbon in the blood, forming carbonic acid gas, which is breathed out.

Thus, oxygen is essential to life, and any material reduction of its proportion in the atmosphere is ruinous to health, and ultimately destroys life. By breathing and rebreathing the air of a closed room, large quantities of its oxygen are consumed, while carbonic acid gas takes its place ; emanations from the bodies and clothing of people add their impurities ; and frequently these are supplemented with the fumes of liquor and tobacco.

Whoever has entered a railroad car at a way station, on a cold day, can never forget the peculiar flavor of the odors that greeted his nostrils, as he opened the door and looked in upon forty or fifty passengers, each one of whom had perhaps occupied the car long enough to have inflated his lungs several times, with the same vitiated air that had done similar service for each of his fellow-travelers. Yet he would be a bold man who would venture to improve the condition of this atmospheric cess-pool, by opening a door or window. He would be certain to excite the anger, perhaps, of the most intelligent men—in appearance—in the car. We should consider that man a lunatic, who, after having taken his bath and changed his linen, would go to the outlet of a sewer and inhale its gases for an hour or two ; but four hundred and fifty thousand of the half-million people who daily go to New

York from the suburbs uncomplainingly submit to treatment that is quite equivalent when they travel in cars and ferry-boats, because the majority of passengers are as indifferent to sanitary laws as the people who manage these conveyances. Were a knowledge of the processes of life, the sources of contamination and the methods of neutralizing them, as well and as generally understood as hundreds of things of far less importance, diphtheria, summer diseases of children, many forms of fever, bronchial troubles and consumption would hardly be known. But men seldom trouble themselves about health till they lose it. Until within a few years country houses in the Northern and Eastern States were usually built with low ceilings, because such rooms were *warmer*. Bedrooms were made small for the same reason ; the single door was kept closed in cold weather and the single window carefully caulked to exclude every breath of air. It was found in due time that farmers—who usually occupied such houses—were the shortest-lived people in the country. This fact was attributed to overwork, until science demonstrated that it was mainly due to blood poisoning, in the manner above stated.

There has been considerable discussion as to whether or not consumption is a contagious disease. It is now well settled that under certain conditions it is ; just as many other diseases are contagious under similar circumstances.

A knowledge of these conditions is of the highest importance to every one. A person in almost perfect health, with no inherited tendency to lung disease, becomes the nurse and almost constant attendant of a consumptive—a wife or child, perhaps. In his desire to keep the patient comfortable—for consumptives are very sensitive to cold—the chamber is kept closed, little regard being had to proper ventilation. The care and anxiety, with the vitiated air, soon produce a marked effect on the attendant ; the vital power is lowered ; he breathes in the seeds of disease ; they find lodgment, and in time he dies of consumption, thus contracted.

The poorest ventilation is in our street cars. The windows are, on some roads, so fastened that they can not be opened except by a carpenter ; the ventilators in the roof are kept constantly closed, and the doors are only opened to let passengers in and out. Next to the street cars, many of the ferry-boats are perhaps the most insalubrious and uncomfortable places that passengers are compelled to encounter.

The passenger cars of our best railroads have tolerable appliances for ventilation, but they are of very little use when most needed. There is always some *public-spirited* individual ready to firmly close all the ventilators as fast as two men could open them. The better plan would be to have openings a foot square over each door, as high up as possible. Those openings might be covered with wire gauze, to exclude cinders. A shield of wood or tin should be fastened inside at the lower part of the opening and reaching upward at an angle of 45 degrees, far enough to conceal the ventilator. This would direct the air upward so that it would not blow directly on the passengers. All air and foul gases at first rise to the ceiling, and they would thus be carried out of the ventilator at the rear of the car. There should be no way of closing these ventilators in Winter or Summer. It would require but little more heat to make the cars comfortable by reason of the ventilators.

In ventilating houses it is important to remember that the bad air first rises to the ceiling, leaving the purest air near the floor; therefore ventilators should be as near the ceiling as possible, and so arranged that all currents of air shall be directed upward. Fresh air invigorates; it is the current of air blowing directly on some part of the body that produces cold, rheumatism, etc. Open fire-places conduct the air rapidly up the chimney; but the trouble is they carry off the pure air first, as they are near the floor. Still such ventilation is far better than none.

Letting down opposite windows from the top gives good ventilation in cold weather, or if there are no windows opposite, an opening high up in the chimney answers an excellent purpose. In Summer it is well to open all doors and windows; but persons should avoid sitting in draughts of air.

---

WE call especial attention to the splendid offer which we now make to subscribers. For $7 we will send the *Church Union* for one year and "Chambers' Encyclopedia," fifteen volumes, a verbatim reprint of the last London edition. There are eight hundred pages in each volume, and the whole comprises a complete "Dictionary of Universal Knowledge," or we will send the Encyclopedia to every one paying $10 for four years subscription in advance, or to any one sending four new subscribers at $2.50 each. Those who have seen the work can scarcely credit the possibility of furnishing it at such a remarkably low price. Address E. B. GRANNIS, publisher *Church Union*, 22 Beekman street, New York City.

